# Uncommon Presentation of a Giant Cell Tumor of the Tendon Sheath of the Hand: A Case Report

**DOI:** 10.7759/cureus.49310

**Published:** 2023-11-23

**Authors:** Daniel A Meza-Martinez, Hugo E Beyuma-Mora, Julio A Palomino-Payan, Brando J Fematt-Rodriguez, Irean Garcia-Hernandez

**Affiliations:** 1 General Surgery, Instituto Mexicano del Seguro Social, Hospital General de Zona No. 33, Monterrey, MEX; 2 Plastic and Reconstructive Surgery, Instituto Mexicano del Seguro Social, Unidad Médica de Alta Especialidad, Hospital de Traumatología y Ortopedia No. 21, Monterrey, MEX; 3 Pathology, Instituto Mexicano del Seguro Social, Unidad Médica de Alta Especialidad, Hospital de Traumatología y Ortopedia No. 21, Monterrey, MEX

**Keywords:** recurrence risk, flexor digitorum superficialis, tendon sheath, finger tumor, tenosynovial giant cell tumor

## Abstract

A giant cell tumor of the tendon sheath (GCTTS) presents as a rare neoplasm demanding a heightened index of suspicion for precise diagnostic evaluation, especially when manifesting in the digital phalanges, as it is part of a group of neoplasms known as tenosynovial giant cell tumors (TCGTs). An accurate and timely diagnosis is crucial, as it significantly enhances treatment outcomes for this heterogeneous group of lesions. We describe the case of a male patient who presented with multiple nodules in the fourth finger of his left hand and was ultimately diagnosed with a localized form of a GCTTS, an unusual presentation for localized forms of this entity. Our objective is to outline the diagnostic and therapeutic approach, discussing options for differential diagnosis and treatment modalities. To achieve this, we conducted a literature review and compared our findings and the observed evolution in our patient. Early recognition of hand tumors allows for timely diagnosis, facilitating optimal resections during surgical procedures. This, in turn, reduces morbidity and enhances the functionality of the affected extremity, as detailed in the current case.

## Introduction

The term tenosynovial giant cell tumor (TCGT) encompasses a group of neoplasms primarily originating in the synovial membrane of joints, bursae, or tendon sheaths, displaying synovial differentiation. These lesions typically manifest either in extremities or in large joints. Localized forms usually appear as a single nodule on the hands, while diffuse forms, seen in large joints such as the knee, often display multiple masses [1]. The estimated annual incidence rate of TGCTs varies depending on their form, ranging from 1.8 to 50 cases per million. Etiological factors considered for these entities include trauma, inflammation, metabolic diseases, and neoplastic origins [1,2]. The primary risks associated with these neoplasms are recurrence and the potential for joint damage leading to altered mobility, which may require non-conservative surgical interventions. Diffuse types of a giant cell tumor of the tendon sheath (GCTTS) tend to exhibit a higher recurrence rate compared to localized forms [3]. We report the case of a male patient with an atypical presentation of a localized GCTTS, as he exhibited multiple firm and small masses on the fourth finger of his left hand, which had been growing for the past four years. He underwent successful treatment with complete resection, preserving all adjacent finger structures. Subsequently, the diagnosis of localized GCTTS was confirmed through histopathological analysis, despite its unusual presentation pattern, as it normally manifests as a single nodule when appearing on the hand.

## Case presentation

A 69-year-old male patient, with a medical history of type 2 diabetes and hypertension, was referred to our outpatient clinic. He presented with multiple nodules on the fourth finger of his left hand that showed progressive and slow growth over the past four years. His main complaint was limited finger mobility, particularly during finger flexion.

**Figure 1:**
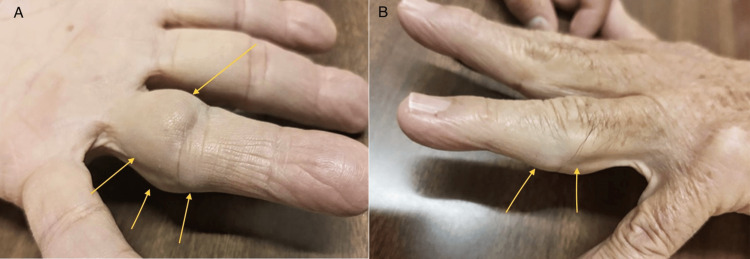
Clinical findings. The patient presented with multiple well-defined rounded tumors located on the fourth finger of his left hand. A: Multiple tumors located in the proximal phalanx of the fourth finger in flexor zone II (yellow arrows). B: Tumoral growth on the ulnar edge of the proximal phalanx (yellow arrows).

Physical examination revealed diminished mobility of the fourth finger during flexion of the metacarpophalangeal joint. He reported no pain during finger movement, and his left hand exhibited normal color, capillary refill, and no sensory deficits. Limitation in flexion of the affected joint was due to the presence of multiple firm, lobulated, and non-tender masses located on the palmar and ulnar aspects of the proximal phalanx of the affected finger, which measured approximately 15 x 10 mm each, they did not coalesce and were partially mobile (Figure 1).

**Figure 2:**
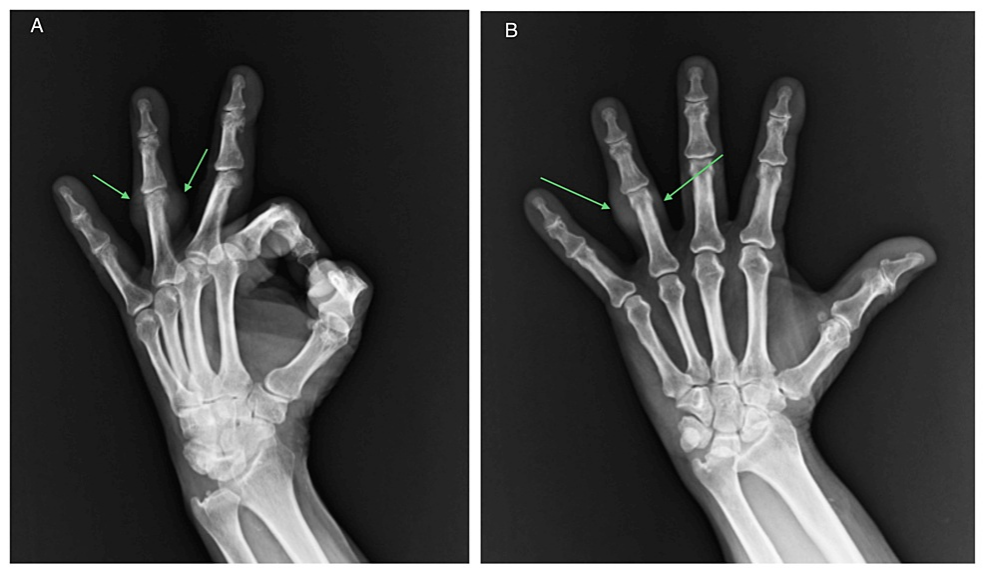
Radiological findings. A soft tissue tumor was identified at the head of the proximal phalanx of the fourth finger and near the proximal interphalangeal joint, with no evidence of bone tissue involvement. A: Lateral projection X-ray of the left hand reveals the soft tissue tumor's location without any evidence of bone erosion. B: Anteroposterior projection that illustrates the tumor's vicinity to the proximal interphalangeal joint.

A plain X-ray examination of the patient's left hand in two projections revealed radiopaque tumors within the soft tissue regions, precisely at the head of the proximal phalanx of the fourth finger and near the proximal interphalangeal joint (Figure 2).

**Figure 3:**
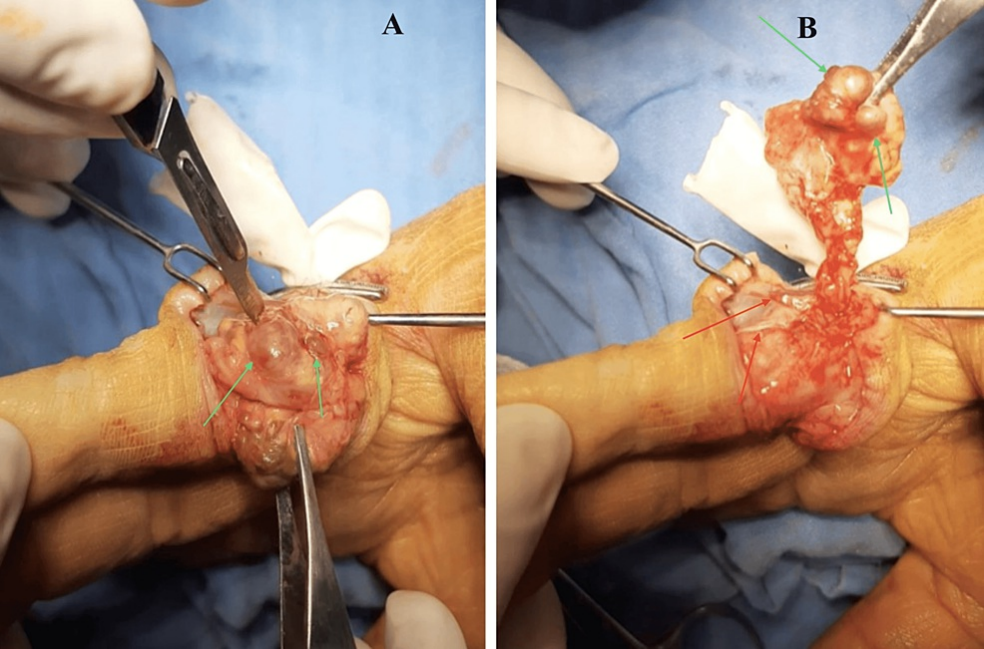
Intra-operative findings and surgical approach. The multilobulated tumor displayed loose adhesions, with minimal attachment to digital anatomical structures, neurovascular bundles, and the flexor tendon sheath. A: Image that displays a multilobulated tumor (green arrows) extending from flexor zone II to extensor zones III and IV. B: Image demonstrating loose adhesions (red arrows) and minimal attachment to digital anatomical structures, neurovascular bundles, and the flexor tendon sheath. Multilobulated tumor indicated by green arrows.

An elective digital exploration was scheduled under local-regional anesthesia based on our clinical and radiological suspicion of a hand tumor, likely a giant cell tumor. The procedure was successfully performed without complications, revealing a multilobulated, firm, and amber-colored tumor. Notably, the tumor extended from the palmar aspect of the hand in flexor zone II to the ulnar and dorsal aspects of extensor zones III and IV. Importantly, the tumor displayed no significant adhesions to digital anatomical structures, and we exercised great care in preserving the integrity of neurovascular bundles, as well as the flexor and extensor apparatus of the finger (Figure 3).

**Figure 4:**
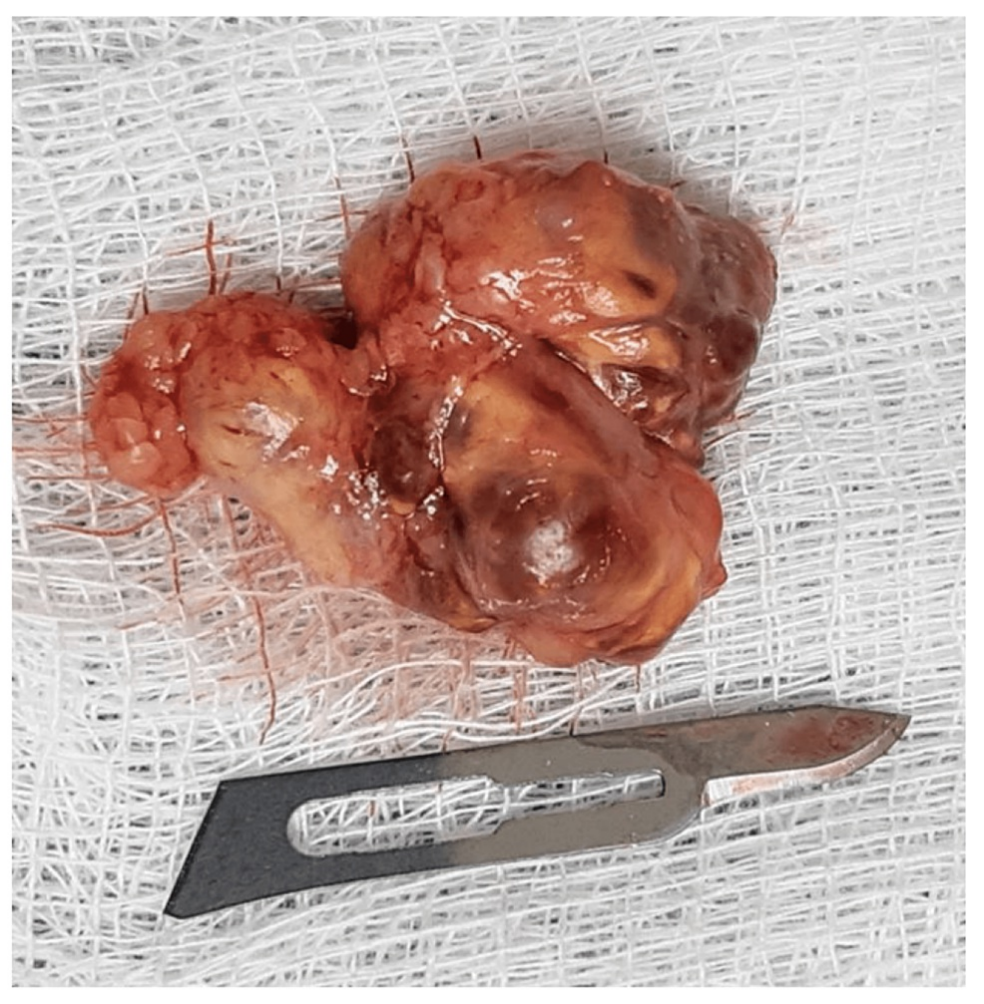
Surgical specimen. A well-circumscribed, partially encapsulated, multilobulated tumor, firm and amber-colored, measuring 35 mm in length and 25 mm in width.

We performed a complete resection (Figure 4) followed by primary closure of the surgical site that required skin remodeling due to tissue expansion secondary to tumoral growth.

**Figure 5:**
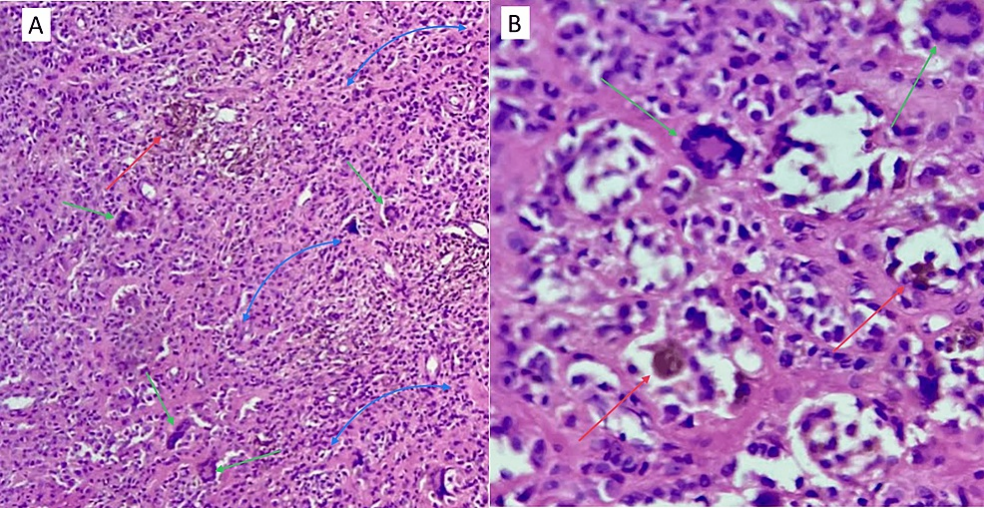
Histopathological examination revealed findings consistent with localized GCTTS. A: 10x. The tumor comprised mononuclear cells (blue arrows) and an abundance of osteoclast-type multinucleated giant cells (green arrows) within a fibrous stroma. Hemosiderin deposits are also shown (red arrows), commonly described in localized forms. There is an absence of atypia, mitosis, or any signs of malignancy. B: 40x. Multinucleated giant cells (green arrows) within a fibrous stroma, accompanied by inflammatory cells, including macrophages with hemosiderin (red arrows). GCTTS: Giant cell tumor of the tendon sheath

In the post-operative follow-up, sutures were removed, revealing satisfactory wound healing and good digital mobility. Subsequent histopathological analysis categorized the specimen as localized GCTTS (Figure 5). The post-operative period was uneventful, with no recurrence observed over an 18-month follow-up. Given the patient's favorable post-operative progress and the histological diagnosis, we decided to discharge the patient from our care.

## Discussion

TGCTs are a group of neoplasms that primarily affect individuals between the ages of 20 and 50. They can manifest as either a single nodule, referred to as a localized TGCT, or as multiple nodules, known as diffuse TGCTs, along a synovial layer, bursa, or tendon sheath. While the TGCT predominantly involves large joints like the knee and hip, it can also affect fingers, and, on rare occasions, the temporomandibular joint or the spine [2]. Localized TGCT forms are consistently benign, while diffuse TGCT forms can be more aggressive and destructive, with rare instances involving a malignant component. This wide spectrum of anatomical and clinical presentations, as well as diverse biological behaviors, contributes to the complexity of therapeutic management [4]. Unlike typical descriptions in the literature, our patient did not present a single tumor and did not fall within the usual age group for these neoplasms, initially raising concerns about malignancy. This case highlights the diverse presentations of tenosynovial tumors, even so within localized forms of GCTTS. In this instance, a hand X-ray played a pivotal role in ruling out malignant tumors, eliminating the necessity for further preoperative imaging studies.

Conventional radiography is often employed as an initial diagnostic tool; however, it may not always detect abnormalities but is used to rule out calcifications, which are infrequently observed in TGCTs but may be present in other potential diagnoses. MRI is the preferred method for detecting and characterizing TGCTs [5,6]. In our case, we found that conventional radiography was the only necessary preoperative study. The absence of radiological signs suggestive of malignancy, such as bone erosions or calcifications, directed our diagnostic suspicion toward a benign tumor. It is worth noting that MRI was not readily accessible at our institution at that time.

Several factors have been associated with recurrence, such as proximity to the distal interphalangeal joints, the presence of degenerative joint disease, pressure erosions in radiographs, increased mitotic activity, and type 2 lesions. However, the one consistent observation made by multiple authors in preventing recurrence is the complete surgical excision, including the removal of all satellite nodules when present [1,7]. The localized nodular type is typically benign, whereas the diffuse type is more invasive and may, on rare occasions, progress to malignancy [8]. The patient had no identifiable risk factors for the development of this condition, as these factors are not well-researched. Similarly, there were no associated risk factors for recurrence, as noted in the literature. In our case, complete tumor resection resulted in an optimal outcome without recurrence, even though the tumor became noticeable four years ago.

Resection serves as the initial treatment choice for TGCTs. The most suitable surgical approach remains a subject of debate and hinges on the tumor's specific location. Additionally, treatment options encompass radiosynovectomy, external beam radiotherapy, chemotherapy, and the use of tyrosine-kinase inhibitors, either in conjunction with surgery or as standalone methods. The localized-type TGCT carries a 10-year recurrence risk of up to 9.8% and a lifetime recurrence risk of up to 15%. In the case of a localized-type TGCT, a simple surgical excision as a standalone treatment is typically sufficient for a cure and can be confirmed during the surgical procedure [5]. Since this was the case of a localized GCTTS, meticulous dissection of all finger components, including the flexor and extensor tendons, neurovascular bundle, and tumor tissue, enabled a complete lesion resection without recurrence or harm to structures that could impair function. The use of loupes and magnifying lenses was instrumental in accurately distinguishing between tissues. With complete resection, no additional treatment was necessary for this patient.

## Conclusions

This case report underscores the importance of clinical suspicion, thorough evaluation, and surgical management in the approach to GCTTS of the hand. Furthermore, it highlights the need for further research to better understand the pathogenesis and associated risk factors of this rare neoplastic entity. Achieving complete resection through timely diagnosis and appropriate surgical treatment in patients with GCTTS not only preserves hand function but also reduces the risk of recurrence, much like what occurred in this patient. Histopathological analysis is essential to differentiate giant cell tumors from entities with malignant potential and other forms of TCGTs.
